# A Review Study on Sulfate-Radical-Based Advanced Oxidation Processes for Domestic/Industrial Wastewater Treatment: Degradation, Efficiency, and Mechanism

**DOI:** 10.3389/fchem.2020.592056

**Published:** 2020-11-27

**Authors:** Xinhui Xia, Fengyi Zhu, Jianju Li, Haizhou Yang, Liangliang Wei, Qiaoyang Li, Junqiu Jiang, Guangshan Zhang, Qingliang Zhao

**Affiliations:** ^1^State Key Laboratory of Urban Water Resources and Environment (SKLUWRE), School of Environment, Harbin Institute of Technology, Harbin, China; ^2^College of Resource and Environment, Qingdao Agricultural University, Qingdao, China

**Keywords:** sulfate-radicals, wastewater, activation approaches, degradation kinetics, reaction mechanisms

## Abstract

High levels of toxic organic pollutants commonly detected during domestic/industrial wastewater treatment have been attracting research attention globally because they seriously threaten human health. Sulfate-radical-based advanced oxidation processes (SR-AOPs) have been successfully used in wastewater treatment, such as that containing antibiotics, pesticides, and persistent organic pollutants, for refractory contaminant degradation. This review summarizes activation methods, including physical, chemical, and other coupling approaches, for efficient generation of sulfate radicals and evaluates their applications and economic feasibility. The degradation behavior as well as the efficiency of the generated sulfate radicals of typical domestic and industrial wastewater treatment is investigated. The categories and characteristics of the intermediates are also evaluated. The role of sulfate radicals, their kinetic characteristics, and possible mechanisms for organic elimination are assessed. In the last section, current difficulties and future perspectives of SR-AOPs for wastewater treatment are summarized.

## Introduction

The ongoing development of industries and agriculture has resulted in increasing discharge of toxic organic contaminants such as pesticides, antibiotics, dyes, and pharmaceuticals into drainage systems and, consequently, into wastewater treatment plants (WWTPs). These toxic organic pollutants have attracted considerable attention recently (Martin-Diaz et al., 2020; Qin et al., 2020b; Seleiman et al., 2020). Owing to the high toxicity, polymeric structures, and non-biodegradable characteristics of these pollutants, traditional biological treatment approaches for WWTPs are not effective as they cannot destroy their structures. The treatment works thus fail to address the ultimate eradication of these substances (Babu et al., 2019). In addition, some of the widely used physical (e.g., filtration, flotation) or chemical (e.g., chemical coagulation and electrochemistry) processes have also been criticized for the high production of solid waste and secondary contamination. There is therefore a need to explore alternatives for effective and environmentally friendly treatment of these organic contaminants, to provide a better approach for degradation of these hazardous wastes before effluent is discharged into aquatic environments.

Advanced oxidation processes (AOPs) are characterized by being easy to implement, highly efficient, environmentally compatible, and capable of oxidizing a wide range of contaminants (mineralization to CO_2_, H_2_O, and inorganic ions). Further, they have been widely applied in refractory contaminant degradation (Huang and Zhang, 2019). Specifically, the generated free radicals, such as hydroxyl radical (OH·), superoxide radicals (${\text{O}}_{2}^{-}\cdot$), and sulfate radicals (${\text{SO}}_{4}^{-}\cdot$), and hole (h^+^) play a significant role in pollutant degradation. However, the application of OH**·**-based AOPs is partially limited because of the necessary acidic environmental conditions (pH 2–4) and H_2_O_2_ instability (Liu et al., 2018b).

Among the AOPs mentioned above, activated persulfate (PS) oxidation has been widely studied recently for a relatively long lifetime (30–40 μs) of the sulfate radicals (${\text{SO}}_{4}^{-}\cdot$, *E*_0_ = 2.5–3.1 V) and under moderate reaction conditions (4 < pH < 9) (Ren et al., 2015). In overall, sulfate radical advanced oxidation processes (SR-AOPs) have the following advantages: (i) higher redox potential of ${\text{SO}}_{4}^{-}\cdot$ (*E*_0_ = 2.5–3.1 V vs. NHE) than that of OH**·** (*E*_0_ = 1.8–2.7 V vs. NHE); (ii) more moderate reactional pH conditions of 2.0–8.0; (iii) longer half-life (*t*_1/2_ = 30–40 μs) (Li et al., 2019a); and (iv) higher oxidation capacity in both carbonate and phosphate buffer solutions (Li et al., 2016; Liu et al., 2018b; Yang Q. et al., 2019). Hence, SR-AOPs are regarded as the most promising advanced oxidation processes for water and wastewater treatments.

Generally, peroxymonosulfate (PMS) or PS can be activated to generate ${\text{SO}}_{4}^{-}\cdot$ radicals via appropriate heating, ultraviolet (UV) irradiation, or transition metal (Fe^2+^, Co^2+^, Ag^+^) activation (Cai et al., 2015; Li et al., 2015; Yang W. et al., 2019). For example, sulfate radicals can be spontaneously produced from PS or PMS via the physical activation methods: (1) heating (Ji et al., 2016b), (2) light radiation (Khan et al., 2017), (3) ultrasonic waves (Deng et al., 2015), and the chemical activation approaches: (4) transition metal ion activation (Chen et al., 2019), (5) alkaline activation (Fernandes et al., 2018), (6) strong oxidizers (M'Arimi et al., 2020), and (7) electrochemistry activation (Li et al., 2019a). Innovative research of SR-AOPs mainly includes (1) new catalytic activation approaches, (2) novel role of free radicals, and (3) new degradation pathways and mechanisms. To further clarify the potential mechanisms of the activation, oxidation, and elimination processes of sulfate radicals, the relevant findings are summarized here.

In this paper, we first summarize the activation methods of PMS and PS for efficient sulfate radical generation. The efficiency of the various activation methods on PS and PMS is then evaluated via feasibility, key parameters, and economic analysis. Third, the possible mechanisms for degradation of organic pollutants using sulfate radicals are described. Finally, the challenges and perspectives for sulfate-radical-based advanced oxidation processes are put forward.

## Sulfate Radical Generation and Efficiency Improvement

### Properties of PMS and PS

Sulfate radicals can be efficiently generated via the activation of PSs, such as peroxydisulfate (PDS, S_2_${\text{O}}_{8}^{2-}$) and permonosulfate (PMS, ${\text{HSO}}_{5}^{-}$) (Wacławek et al., 2017; Yang W. et al., 2019), both of which are characterized by the presence of an O–O bond (similar to hydrogen peroxide). Generally, PMS, a typical compound salt, mainly consists of potassium hydrogen sulfate, potassium sulfate, and potassium peroxymonosulfate at a ratio of 1:1:2 (Wacławek et al., 2017). In contrast, PDS contains sulfate ions and positive ions.

The asymmetric structure of PMS, generated via the substitution of hydrogen atoms in H_2_O_2_ by SO_3_, is characterized by an O–O bond distance of 1.453 Å (Li et al., 2016). In comparison, PDS possesses a longer bond distance (1.497 Å) and lower bond energy (33.5 kcal mol^−1^), which is closely related to the substitution of two individual hydrogen atoms of H_2_O_2_ by SO_3_ (Xiao et al., 2018; Song et al., 2019). Thus, theoretically, the chemical characteristics for PDS are more stable than for PMS; PDS also has a higher redox potential than PMS (2.01 vs. 1.77 V) (Wacławek et al., 2017; Xiao et al., 2020). Overall, both PDS and PMS have been widely applied, owing to their excellent water solubility, low cost, ease of storage, and low environmental impact (Xiao et al., 2020). Traditionally, PS refers to PDS, including Na_2_S_2_O_8_, K_2_S_2_O_8_, and (NH_4_)_2_S_2_O_8_ (Tsitonaki et al., 2010; Li et al., 2019b). Among them, Na_2_S_2_O_8_ has been frequently applied because of its lower cost, stable chemical characteristics, and ease of solubility.

### Activation Approaches

**Figure 1:**
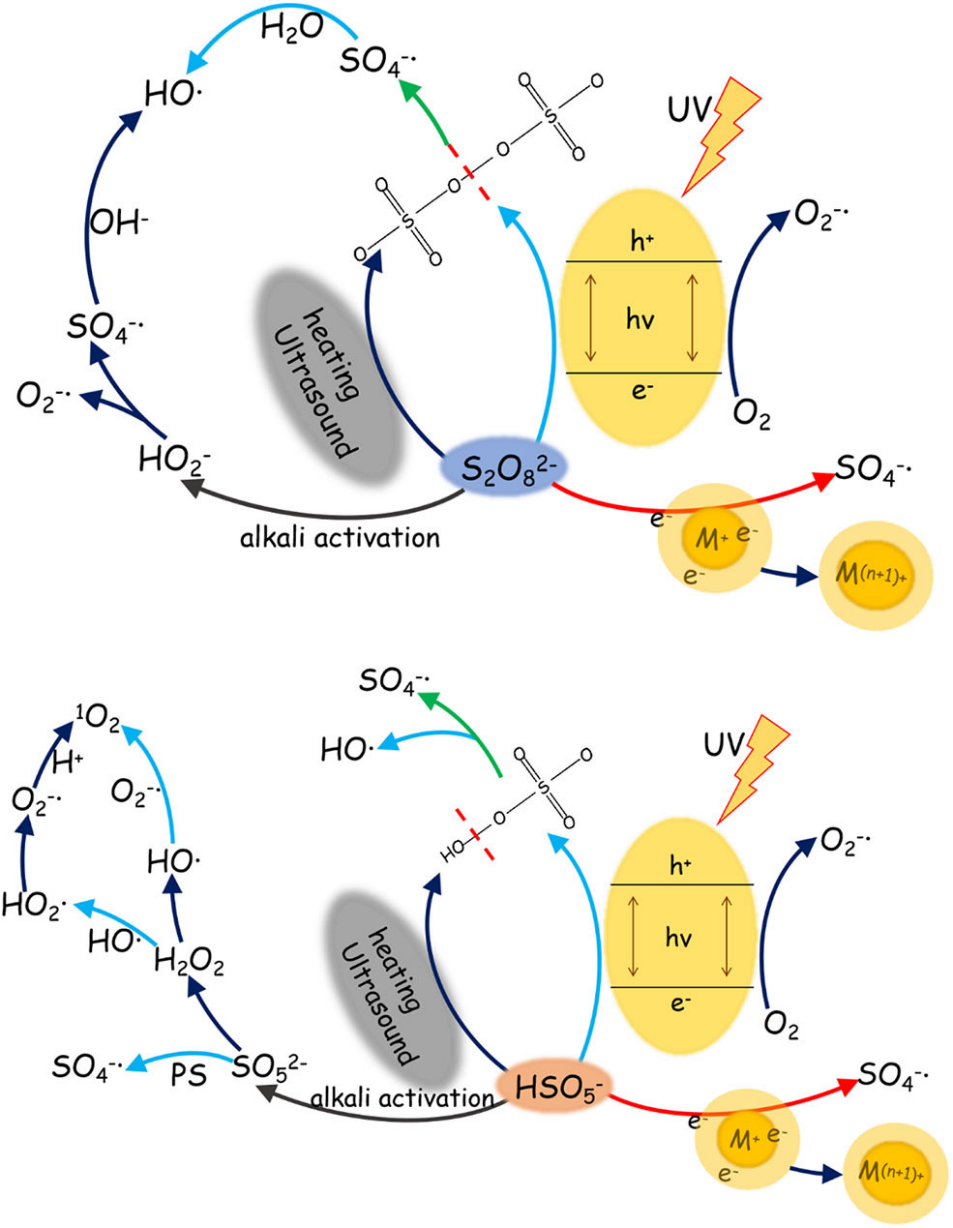
Generation of sulfate radical via persulfate/peroxymonosulfate (PS/PMS) activation under different reaction conditions.

The activation of PS and PMS is mainly conducted through thermal treatment, UV radiation, use of alkaline, and metal ion reactions. There is a wide variation between sulfate radicals produced by different activation approaches of the PS and PMS, in terms of activation mechanisms, pathways, and redox potential (see Table 1). Thus, summarizing the efficiency of different approaches of PS and PMS is fundamental to listing the critical targets of generation of sulfate radicals (see Figure 1).

**Table 1:** Summary of the different persulfate (PS) and peroxymonosulfate (PMS) activation approaches: pathways, mechanism, and key parameters.

**Activation approaches**		**Main reaction for sulfate radicals production**	**Mechanism**	**Key parameters**	**References**
Physical activation	Heating	$S_{2}{O_{8}}^{2-}+\;heat\;\to \;2SO_{\text{4}}^{\text{-}}\;\cdot \;$ (PS) $HS{O_{5}}^{-}+\;heat\;\to \;SO_{\text{4}}^{\text{-}}\;\cdot \;+HO\cdot$ (PMS)	Fission of O–O bond	(i) Higher temperature can cleave O–O bond, whereas excessive temperature may cause side effects (ii) Owing to huge energy demand, not suitable for large-scale sewage treatment	Zhao et al., 2013; Wang and Wang, 2018
	UV	$S_{2}{O_{8}}^{2-}+\;h\nu \;\to \;2SO_{\text{4}}^{\text{-}}\;\cdot \;$ (PS) $HS{O_{5}}^{-}+\;h\nu \to SO_{\text{4}}^{\text{-}}\;\cdot \;+HO\cdot$ (PMS)	Fission of O–O bond	Usually under 254 nm wavelength, depending on dissolved oxygen concentration	Yang Q. et al., 2019
	Ultrasound	$S_{2}O_{8}^{2-}+2H_{2}O\overset{OH^{-}}{\underset{}{\to }}HO_{2}^{-}+2SO_{4}^{2-}+3H^{+}$ $S_{2}O_{8}^{2-}+2HO_{2}^{-}\to SO_{4}^{-}\cdot +SO_{4}^{2-}+H^{+}+O_{2}^{-}\cdot$ (PS)	Fission of O–O bond Hydrolysis of water molecules	Similar to heating activation	Nasseri et al., 2017
Chemical activation	Alkaline activation	$S_{2}O_{8}^{2-}+H_{2}O\to HO_{2}^{-}+2SO_{4}^{2-}+H^{+}$ $S_{2}O_{8}^{2-}+HO_{2}^{-}\to SO_{4}^{-}\cdot +SO_{4}^{2-}+H^{+}+O_{2}^{-}\cdot$ (PS) $HSO_{5}^{-}\to SO_{5}^{2-}+H^{+}$ $HSO_{5}^{-}+H_{2}O\to HSO_{4}^{-}+H_{2}O_{2}$ $SO_{5}^{2-}+H_{2}O\to SO_{4}^{2-}+H_{2}O_{2}$ $H_{2}O_{2}\to HO_{2}^{-}+H^{+}$ *H*_2_*O*_2_ → 2*OH*· $H_{2}O_{2}+OH\cdot \to HO_{2}^{}\cdot +\;H_{2}O$ $HO_{2}^{-}\cdot \to H^{+}+O_{2}^{-}\cdot$ $HO\cdot +O_{2}^{-}\cdot \to^{1}O_{2}+OH^{-}$ $2H^{+}+2O_{2}^{-}\cdot \to^{1}O_{2}+H_{2}O_{2}$ $HSO_{5}^{-}+SO_{5}^{2-}\to HSO_{4}^{-}+SO_{4}^{-}\cdot {+}^{1}O_{2}$ (PMS)	Hydrolysis of PS and PMS to hydrogen peroxide	Often pH > 11 For PMS activation, ^1^O_2_ is the predominant radical specie, whereas SO${}_{4}^{-}\cdot$/OH·/^1^O_2_ for PS	Fernandes et al., 2018
	Transition metal ions	$S_{2}O_{8}^{2-}+M^{n+}\to M^{n+1}+SO_{4}^{2-}+SO_{4}^{-}\;\cdot \;$ (PS) $HSO_{\text{5}}^{-}+M^{n+}\to M^{n+1}+OH^{\text{-}}+SO_{4}^{-}\;\cdot \;$ (PMS)	Single electron transfer	Preparation of catalysts is more economical for a homogeneous system than that of a heterogeneous system	Liu L. et al., 2019
	Carbon-based materials	$S_{\text{2}}O_{\text{8}}^{2-}+ACsurface-OOH\to SO_{4}^{-}\cdot \;+ACsurface-OO\cdot \;+HSO_{4}^{-}$ $S_{\text{2}}O_{\text{8}}^{2-}+ACsurface-OH\to SO_{4}^{-}\cdot \;+ACsurface-O\cdot \;+HSO_{4}^{-}$ (PS) $HSO_{5}^{-}+C-\pi \to C-\pi^{+}+SO_{4}^{-}\cdot \;+OH^{-}$ $HSO_{5}^{-}+C-\pi^{+}\to C-\pi +SO_{5}^{-}\cdot \;+H^{+}$ (PMS)	Single electron transfer	Activated carbon is relatively economical	Zhao et al., 2017

#### Physical Activation Approaches

##### Thermal Activation

Thermolysis has traditionally been recognized as one of the most effective approaches for activating PS/PMS for ${\text{SO}}_{4}^{-}$· generation (Equations 1 and 2) (Zhou et al., 2019), despite intensive energy demand. The recent work of Ji et al. (2016b) showed that the ambient temperature (20 ± 1°C) could not activate PS efficiently for the purpose of tetracycline (TTC) oxidization, whereas there was complete elimination of TTC at 70°C after 30 min reaction under acidic conditions. This clearly demonstrated that high temperature promotes the formation of sulfate radicals. A similar result was also obtained by Zhou et al. (2019), who observed a significant enhancement of PS decomposition and ${\text{SO}}_{4}^{-}$ generation as the temperature increased from 20 to 75°C. Simultaneously, a noteworthy increase in acidic by-product formation was also observed (Zhou et al., 2019). For soil samples, Deng et al. (2015) found that increased temperature promoted PS oxidization of phenanthrene. However, Tsitonaki et al. (2010) found that high temperatures can negatively affect the transport of PS into soil; they can further affect the overall removal behavior of organics in soil (Zhao et al., 2013). Overall, thermal activation is not applicable to PS/PMS activation for large-scale soil remediation (Wang and Wang, 2018).

(1) $$\begin{array}{l} S_{2}{O_{8}}^{2-}+heat\to 2SO_{4}^{-}\cdot \end{array}$$

(2) $$\begin{array}{l} HS{O_{5}}^{-}+heat\to SO_{4}^{-}\cdot +HO\cdot \end{array}$$

Fission of the O-O bond of PS/PMS at temperatures > 50°C (energy input 140.0–213.3 kJ/mol) during heating activation plays an essential role in the formation of sulfate radicals (Wang and Wang, 2018). Meanwhile, the generated sulfate radicals would quickly be converted to hydroxyl radicals and would subsequently cause a decrease in pH of the aqueous solution (Yang et al., 2010) (Equation 3). In addition, Yang et al. (2010) reported that the degradation efficiency of the heat-activated PS was higher than that of PMS, which may be attributed to the higher O-O bond energy of PMS than that of PS.

(3) $$\begin{array}{l} SO_{4}^{-}\cdot +H_{2}O\to S{O_{4}}^{2-}+HO\cdot +H^{+} \end{array}$$

##### Ultraviolet Activation

Ultraviolet irradiation has been regarded as one of the most efficient approaches for PS/PMS activation because of the high irradiation energy of UV. The activation of PS/PMS under UV light is generally related to the cleavage of the O–O bond. Two ${\text{SO}}_{4}^{-}\cdot$ could be generated after the activation of PS (Equation 4), whereas OH**·** and ${\text{SO}}_{4}^{-}\cdot$ are generated through PMS activation because of its asymmetric structure (Equation 5). Similar to heat activation, some of the generated ${\text{SO}}_{4}^{-}$ could be further transformed to OH**·** through UV activation (Equation 6) (related to the pH of the aqueous solution).

(4) $$\begin{array}{l} S_{2}{O_{8}}^{2-}+h\nu \to 2SO_{4}^{-}\cdot \end{array}$$

(5) $$\begin{array}{l} HS{O_{5}}^{-}+h\nu \to SO_{4}^{-}\cdot +HO\cdot \end{array}$$

(6) $$\begin{array}{l} SO_{4}^{-}\cdot +H_{2}O\to S{O_{4}}^{2-}+HO\cdot \end{array}$$

The work of Chen et al. (2019) found that ${\text{SO}}_{4}^{-}\cdot$ was the predominant radical under the condition of pH < 7.0, whereas ${\text{SO}}_{4}^{-}\cdot$ would convert to OH^−^ once the pH increased to 9.3 (Ao et al., 2018). Regarding different organic contaminants, UV/PS and UV/PMS have recently been employed for refractory organics degradation. Many studies have demonstrated that UV/PS shows a stronger oxidizing capacity for organic pollutants than UV/PMS, closely related to the higher quantum yielding (Carlson et al., 2015). However, UV merely accounts for a small fraction of solar light (3–5%); hence, the wavelength range for photoactivation of PS and PMS has recently been expanded from UV to visible light and even to solar light (Guo et al., 2018; Zhu et al., 2020).

##### Ultrasound Activation

The main mechanisms of the ultrasonic activation of PS/PMS (related to cavitation bubbles) can be summarized as follows: (1) ultrasonic generation of sulfate radicals via homolysis of the O–O bond at high temperatures (5,000 K) and pressures (similar to the energy-induced mechanism of heat and UV activation) (Matzek and Carter, 2016; Wang and Wang, 2018) and (2) generation of hydroxyl radicals and hydrogen radicals via the decomposition of water molecules under the action of cavitation bubbles (Equation 7). The recent work of Wang and Wang (2018) found that the yield of hydroxyl radicals was much higher than that of sulfate radicals under ultrasonic activation, especially for PS (Wang and Wang, 2018). However, the recombination rate of hydroxyl radicals was suppressed at high temperatures, owing to the low dissociation energy of the O–O bond of H_2_O_2_. Thus, the concentration of hydroxyl radicals can reach the millimolar level under ultrasonic conditions (von Sonntag, 2008).

(7) $$\begin{array}{l} H_{2}O\to HO\cdot \text{+}H\cdot \end{array}$$

Where the symbol “~” represents ultrasonic range.

Tremendous works demonstrated that the ultrasound activation of PS was more suitable for sulfate radical generation at ambient temperature (Nasseri et al., 2017), and the increase in temperature would negatively affect its efficiency, especially when the temperature reached 65°C (Deng et al., 2015). Similar to heating activation, ultrasound still has the disadvantages of high cost and huge energy consumption. Moreover, ultrasound activation of PS can be easily achieved under alkaline conditions (pH > 10), and a tremendous amount of ${\text{SO}}_{4}^{-}$, superoxide (${\text{O}}_{2}^{-}$), and OH^−^ is generated (Equations 8–10) (Nasseri et al., 2017).

(8) $$\begin{array}{l} S_{2}O_{8}^{2-}+2H_{2}O\overset{OH-}{\to }HO_{2}^{-}+2SO_{4}^{2-}+3H^{+} \end{array}$$

(9) $$\begin{array}{l} S_{2}O_{8}^{2-}+2HO_{2}^{-}\to SO_{4}^{-}\cdot +SO_{4}^{2-}+H^{+}+O_{2}^{-}\cdot \end{array}$$

(10) $$\begin{array}{l} SO_{4}^{-}\cdot +OH^{-}\to SO_{4}^{2-}+OH\cdot \end{array}$$

#### Chemical Activation Approaches

##### Alkaline Activation

Alkali activation of PS/PMS, one of the most widely used *in situ* approaches, involves increasing pH (usually pH > 11) via addition of sodium or potassium hydroxide (Matzek and Carter, 2016; Wacławek et al., 2017). Hydrolysis of one PS molecule to a hydrogen peroxide anion (${\text{HO}}_{2}^{-}$) and, subsequently, to superoxide radicals (${\text{O}}_{2}^{-}$) plays a key role in the activation process (Equations 11 and 12) (Matzek and Carter, 2016; Wacławek et al., 2017; Wang and Wang, 2018).

(11) $$\begin{array}{l} S_{2}O_{8}^{2-}+H_{2}O\to HO_{2}^{-}+2SO_{4}^{2-}+H^{+} \end{array}$$

(12) $$\begin{array}{l} S_{2}O_{8}^{2-}+HO_{2}^{-}\to SO_{4}^{-}\cdot +SO_{4}^{2-}+H^{+}+O_{2}^{-}\cdot \end{array}$$

Moreover, sulfate radicals can further be converted to hydroxyl radicals under alkaline conditions (Equation 13) (Wang and Wang, 2018).

(13) $$\begin{array}{l} SO_{4}^{-}\cdot +OH^{-}\to SO_{4}^{2-}+OH\cdot \end{array}$$

For PMS, alkaline activation exhibits quite a different pathway. As demonstrated by Qi et al. (2016), singlet oxygen and superoxide anion radicals are the predominant reactive oxygen species within the alkali/PMS system. Specifically, hydroxyl radicals could be generated via the hydrolysis of ${\text{HSO}}_{5}^{-}$ (Equations 14–18).

(14) $$\begin{array}{l} HSO_{5}^{-}\to SO_{5}^{2-}+H^{+} \end{array}$$

(15) $$\begin{array}{l} HSO_{5}^{-}+H_{2}O\to HSO_{4}^{-}+H_{2}O_{2} \end{array}$$

(16) $$\begin{array}{l} SO_{5}^{2-}+H_{2}O\to SO_{4}^{2-}+H_{2}O_{2} \end{array}$$

(17) $$\begin{array}{l} H_{2}O_{2}\to HO_{2}^{-}+H^{+} \end{array}$$

(18) $$\begin{array}{l} H_{2}O_{2}\to 2OH\cdot \end{array}$$

Subsequently, the abovementioned hydroxyl radical would further react with excessive hydrogen peroxide and generate superoxide anion radicals (Equations 19 and 20). The hydroxyl radical and superoxide anion radical would then be converted to singlet oxygen and hydroxide ions (Equation 21). In contrast, the superoxide anion radical can also generate hydrogen peroxide and singlet oxygen (Equation 22) (Qi et al., 2016). In addition, singlet oxygen can be generated via self-decomposition (Equation 23) (Wang and Wang, 2018).

(19) $$\begin{array}{l} H_{2}O_{2}+OH\cdot \to {HO}_{2}\cdot +H_{2}O \end{array}$$

(20) $$\begin{array}{l} HO_{2}^{-}\cdot \to H^{+}+O_{2}^{-}\cdot \end{array}$$

(21) HO·+O2-·→O21+OH-

(22) 2H++2O2-·→O21+H2O2

(23) $$\begin{array}{l} HSO_{5}^{-}+SO_{5}^{2-}\to HSO_{4}^{-}+SO_{4}^{-}\cdot {+}^{1}O_{2} \end{array}$$

Compared to that of heating, UV, and metal activations, the alkali-activated PS/PMS has the disadvantages of low efficiency and long degradation time (Crimi and Taylor, 2007; Huling et al., 2011). In addition, Zhao et al. (2013) found that the alkali-activated PS was not significant when used to degrade polycyclic aromatic hydrocarbons.

##### Transition Metal Ions and Metal Oxides

Besides the methods outlined above, PS and PMS can also be activated through transfer of an electron, using metals including cobalt, silver, manganese, and iron, for the formation of sulfate radicals (Equations 24 and 25 where M represents typical metal ions).

(24) $$\begin{array}{l} S_{2}O_{8}^{2-}+M^{n+}\to M^{n+1}+SO_{4}^{2-}+SO_{4}^{-}\cdot \end{array}$$

(25) $$\begin{array}{l} HSO_{5}^{-}+M^{n+}\to M^{n+1}+OH^{-}+SO_{4}^{-}\cdot \end{array}$$

Yun et al. (2019) found that cobalt ions (Co^2+^) could be applied as a catalyst for PMS activation for paracetamol degradation. Similarly, Ji et al. (2016a) showed that the generation of sulfate radicals within the Co(II)/PMS system could be applied to the degradation of tetrabromobisphenol A. The main reaction can be summarized as follows (Equation 26) (Hu and Long, 2016):

(26) $$\begin{array}{l} HSO_{5}^{-}+Co^{2+}\to Co^{3+}+OH^{-}+SO_{4}^{-}\cdot \end{array}$$

The main concern about using cobalt for PMS activation is its potential biological toxicity if used in substantial quantities. Thus, graphene, activated carbon, and metal oxides (Al_2_O_3_, TiO_2_, MgO, etc.) have been widely applied to improve cobalt loading. Shi et al. (2012) proved that, compared with Co_3_O_4_, the use of Co_3_O_4_/GO for PMS activation significantly reduced cobalt leaching. For PS, silver ions are usually selected as one of the most efficient catalysts among the homogeneous metal ions and oxides (Zhou et al., 2018). Important research has demonstrated that the efficiency of PS activation is closely related to increased silver ion loading (Wang J. et al., 2017; Park et al., 2018; Ouyang et al., 2020) (Equation 27).

(27) $$\begin{array}{l} S_{2}O_{8}^{2-}+Ag^{+}\to Ag^{2+}+SO_{4}^{2-}+SO_{4}^{-}\cdot \end{array}$$

Fe ions and their oxides have also been widely studied, owing to their environmentally friendly, non-toxic, and low-cost characteristics (Wei et al., 2020). For example, Fe^2+^-activated PS and PMS have been proven to be one of the most efficient pathways for sulfate radical generation (Equations 28 and 29) (Xiao et al., 2020).

(28) $$\begin{array}{l} S_{2}O_{8}^{2-}+Fe^{2+}\to Fe^{3+}+SO_{4}^{2-}+SO_{4}^{-}\cdot \end{array}$$

(29) $$\begin{array}{l} HSO_{5}^{-}+Fe^{2+}\to Fe^{3+}+OH^{-}+SO_{4}^{-}\cdot \end{array}$$

However, a large dose of Fe^2+^ results in a significant decline in the efficiency/scavenging ability of sulfate radicals (Equations 30) (Rastogi et al., 2009).

(30) $$\begin{array}{l} SO_{4}^{-}\cdot +Fe^{2+}\to Fe^{3+}+SO_{4}^{2-} \end{array}$$

The use of metal ions in homogeneous systems for the activation of PS has the following limitations: (1) low recovery efficiency of metal ions; (2) restriction of reaction pH, with some of the metal ions always precipitating under basic conditions; and (3) organics within the systems negatively influencing the activation of PS and PMS. The abovementioned difficulties can partially be overcome in heterogeneous systems via a combination of metal ions with supports (such as metal oxide, molecular sieve, and carbonaceous material supports) (Xiao et al., 2020).

##### Carbon-Based Materials

*C*arbon-based materials, including activated carbon, graphene, carbon nanotubes, and biochar, have also recently been investigated for PS and PMS activation. The corresponding mechanisms of carbon-based materials in the formation of sulfate radicals, which usually act as an electron donor to PS or PMS, can be described by Equations (31)–(32):

(31) $$\begin{array}{l} S_{2}O_{8}^{2-}+e^{-}\to SO_{4}^{2-}+SO_{4}^{-}\cdot \end{array}$$

(32) $$\begin{array}{l} HSO_{5}^{-}+e^{-}\to SO_{4}^{-}\cdot +OH^{-} \end{array}$$

In addition, the wide distribution of the catalytic moieties, such as quinone, carbonyl, and carboxyl groups, enhances the activation of PS and PMS via the formation of delocalized Π-electrons. The potential mechanism of PMS by carbon-based materials can be summarized in Equations (33)–(34). The corresponding mechanisms of electron transfer from oxygen functional groups are shown in Equations (35)–(36).

(33) $$\begin{array}{l} HSO_{5}^{-}+C-\pi \to C-\pi^{+}+SO_{4}^{-}\cdot +OH^{-} \end{array}$$

(34) $$\begin{array}{l} HSO_{5}^{-}+C-\pi^{+}\to C-\pi +SO_{5}^{-}\cdot +H^{+} \end{array}$$

(35) $$\begin{array}{l} S_{2}O_{8}^{2-}+ACsurface-OOH\to SO_{4}^{-}\cdot +ACsurface-OO\cdot +HSO_{4}^{-} \end{array}$$

(36) $$\begin{array}{l} S_{2}O_{8}^{2-}+ACsurface-OH\to SO_{4}^{-}\cdot +ACsurface-O\cdot +HSO_{4}^{-} \end{array}$$

Carbon nanotubes and graphene were recently applied to the activation of PS and PMS, to enhance efficiency. This is based on the textural structure and surface chemical properties of the carbon nanotubes and graphene during heteroatom doping. The recent work of Pan et al. (2018) found that the catalytic performance of carbonaceous materials improved significantly after nitrogen doping of the carbon nanotubes and graphite oxide. In addition, pyrolysis is widely applied to enhance the performance of carbon-based materials; for instance, the activation performance of biochar is significantly influenced by pyrolysis temperature and time (Zhao et al., 2017).

#### Coupling Activation Approaches

To further enhance the efficiency of activation using the abovementioned systems (heating, UV, alkaline and transition metal ions), hybrid activation systems have been promoted. For instance, cobalt-based catalysts (mentioned in the *Ultrasound Activation* section) exhibited a relatively high PMS activation performance; however, their poor thermal stability restricted their efficiency. Hence, combining cobalt-based catalysts with carbon materials, which provide more surface activation sites, has been widely applied (Bao et al., 2020). Recently, Amor et al. (2019) found that for winery wastewater treatment, the bulk removal efficiency of organics was much greater when using the heat/transition metal ions/S_2_${\text{O}}_{8}^{2-}$ process, compared with the heat/S_2_${\text{O}}_{8}^{2-}$ system. Similarly, ultrasound can induce heating/PS, thus enhancing their performance (Deng et al., 2015). Furthermore, Hu et al. (2020) inferred that microwave and Fe_3_O_4_ had a synergetic effect on PS activation (Hu et al., 2020).

### Economic Analysis of Different Activation Methods for PS/PMS

Electricity/energy consumption (electricity consumed during the operation of the instruments and equipment) and reagent costs (such as catalytic and oxidation reagents) are the two main costs associated with PS/PMS activation processes. Theoretically, physical activation methods, such as heating, UV, and ultrasonic methods, are relatively economical compared with chemical activation, where the latter incur costs due to reagent consumption. However, physical activation approaches are less applicable in large-scale sewage treatment because of the high energy demand. Taking Fe(II)–PS/PMS systems as an example for organic compound removal from wastewater, only 0.47€ was needed per liter for Fe(II)-activated PS, whereas 4.40€ was needed for UV activation. Although carbon-based materials could be a promising activation method for PS/PMS, their high manufacturing cost restricts their use (even though most biochar is made from wood, sludge, and straw agricultural waste). Hybrid activation always costs more than the other approaches, despite having a higher activation efficiency; thus, further research effort should focus on how to balance activation efficiency and cost.

## Oxidation Efficiency of PS/PMS for Contaminant Degradation

Refractory contaminants such as pharmaceutical and personal care products (PPCPs), endocrine disruptors (EDCs), disinfection by-product precursors (DBPs), and high-concentration organic matter (HCOM) are abundant in aquatic environments; they seriously threaten human and ecosystem health. Sulfate-radical-based AOPs, which exhibit excellent efficiency in refractory organic pollutant removal, have recently been applied in the treatment of these contaminants. Because the PS/PMS oxidation systems have proven to be non-selective, degradation performance, efficiency, and possible intermediates are summarized here.

### Performance of PS/PMS Oxidation

The degradation of antibiotics, DBPs, and EDCs within the PS/PMS system has been found to be hot spots requiring research attention, and their rate of degradation was recently used to efficiently evaluate their oxidation performance (Wang Q. et al., 2017; Amor et al., 2019; Qin et al., 2020a). Table 2 shows that the degradation efficiency of the different pollutants is closely related to their chemical species/characteristics and the reaction conditions. Generally, the conditions such as temperature, pH, catalyst loading, and concentration of PS/PMS affect the removal rate of substances. For instance, the degradation efficiency of bisphenol A (BPA) was shown to increase gradually (in a linear relationship) from 46.7 to 66.4 to 76.8 to 96.4 and 99.0% with the PS concentrations increased from 7.4 to 15 to 22 to 30 and 35 mM (Xu et al., 2019). However, the degradation rate of ketoprofen (KET) increased from 69.4 to 97.3 to 100% when the pH gradually increased from 3 to 7 to 10, respectively (Feng et al., 2017).

**Table 2:** Performance of typical pollutants under different reaction conditions.

**Pollutants**		**Reaction systems**	**Performances**	**References**
PPCPs	Carbamazepine (CBZ)	LaCoO_3_/PMS	• LaCoO_3_ calcinated at 600°C showed the best performance • 91.8% of carbamazepine (100 mg/L) was removed within 30 min at pH 6–8 • First-order kinetics	Guo et al., 2020
	Levofloxacin hydrochloride (LVF)	CoFeO_2_@CN/PMS	• CoFeO_2_@CN/PMS performed better than CoFeO_2_@CN and PMS • 89.4% of LVF (10 mg/L) was removed in 20 min using 0.2 g/L catalyst and 0.5 mM PMS	Pi et al., 2020
	Ibuprofen (IBP)	N-doped graphene aerogel (NGA)/PMS	• Catalytic activity increase exhibited NGA>NrGO>GA • 100% of 20 mg/L IBP was removed in 60 min at 45°C • Pseudo-first-order kinetics with activation energy of 87.5 kJ/mol	Wang et al., 2019
	Sulfachloropyridazine (SCP)	Ni@NPC/PS	• Catalytic activity of Ni@NPC>GO>N-rGO>MWCNTs • 100% of SCP (>99%) was removed in 30 min at 45°C	Kang et al., 2019
	Ketoprofen (KET)	Heat/PS	• 100% of 10 μM KET was removed in 60 min at pH = 3 • 97.3% of 10 μM KET was removed in 60 min at pH = 7 • 69.4% of 10 μM KET was removed in 60 min at pH = 10 • Pseudo-first-order kinetics with activation energy of 169.74 kJ/mol	Feng et al., 2017b
	Caffeine (CAF)	Co-MCM41/PMS	• Co-MCM41/PMS performed better than CoO/PMS or Co_3_O_4_/PMS • 100% of 0.05 mM CAF was removed in 20 min using 200 mg/L catalyst and 0.2 mM PMS	Qi et al., 2013
	Diclofenac (DCF)	BFO/PMS	• 65.4% of 0.025 mM DCF was removed in 60 min at pH 3.0 using 0.5 mM PMS and 0.3 g/L BFO • Following two separate concurrent pseudo-first-order catalytic process with different decay rates	Han et al., 2020
	Sulfonamides (SAs)	Heat/PS	• Conditions: reaction time 3 h, reaction temperature 60°C, 2 mM PS and 30 μM SAs, the removal rate of SMX, SIX, STZ, and SMT were 98, 100, 97, and 81%, respectively • Pseudo-first-order kinetics with the activation energy of 109.8 ± 14.7, 133.9 ± 9.5, 130.8 ± 1.1, and 106.2 ± 3.5 kJ/mol for SMX, SIX, STZ, and SMT, respectively	Zhou et al., 2019
EDCs	Bisphenol A (BPA)	Magnetic CFA/PS	• 76.9% of 0.22 mmol/L BPA was removed at 20°C and pH 5 in 60 min using 2 g/L magnetic CFA and 22 mM PS • Pseudo-first-order kinetics	Xu et al., 2019
			• 76.5% of 0.22 mmol/L BPA was removed at 20°C and pH 5 in 60 min using 2 g/L magnetic CFA and 22 mM PS • Pseudo-first-order kinetics	
			• 70.2% of 0.22 mmol/L BPA was removed at 20°C and pH 5 in 60 min using 2 g/L magnetic CFA and 22 mM PS • Pseudo-first-order kinetics	
	Bisphenol A (BPA)	CoS@GN-60/PMS	• 92% of 20 mg/L BPA was removed at pH 6.65 in 8 min using 0.1 g/L catalysts and 0.1 g/L PMS • First-order kinetics	Zhu et al., 2019
	Bisphenol F (BPF)	Sr_2_FeCoO_6_/PMS	• Catalytic activity of Sr_2_FeCoO_6_>SrCoO_3_>SrFeO_3_ • 100% of 20 mg/L BPF was removed at 55°C using 0.45 g/L Sr_2_FeCoO_6_ and 0.2 mM PMS • Pseudo-first-order kinetics	Hammouda et al., 2018
	Tetrabromobisphenol A (TBBPA)	Co(II)/PMS	• More than 96% of 9.2 μM TBBPA was removed at 20°C and pH 8.0 using 0.5 μM Co(II) and 0.2 mM PMS • Pseudo-first-order kinetics	Ji et al., 2016a
DBPs	Iohexol	Co(II)/PMS	• Almost 100% of 50 μM iohexol was removed in 30 min at 25°C and pH 7.0 using 4 mM PMS • Pseudo-first-order kinetics	Zhao et al., 2019
	Bromide	UV/PMS	• 100% of bromide (initial concentration of 20 μM) was removal in 20 min at pH 7.0 and UV intensity 2.19 μE L^−1^ s^−1^ using ≥300 μM PS • Follow pseudo-zero-order kinetics	Fang and Shang, 2012
HCOMs	Metolachlor (MET)	Co(II)/PMS	• 100% of 10 mg/L MET was removed in 40 min at 25°C and pH 6.5 using 0.2 g/L CoFe_2_O_4_ and 3 mM PMS	Liu C. et al., 2019
	Clopyralid (CLP)	Heat/PS	• The removal rate of CLP increased with increasing PS concentration • 57 and 74% of CLP were removed at 50°C when the initial PS concentration was 1.0 and 2.0 mM, respectively	Yang et al., 2020
	Landfill leachate	Heat/PS	• Acidic condition favored persulfate oxidation of leachate organics • High persulfate dose (PS:12COD_0_) and high temperature enhanced the removal of COD and ammonia nitrogen • 100% of ammonia nitrogen was removed at 50°C and pH 5.3 when persulfate dose was 2 • 91% of COD was removed at 50°C and pH 4 when persulfate dose was 2	Deng and Ezyske, 2011

On the other hand, the catalyst activity was shown to significantly influence the degradation of contaminants; this could be proven by the fact that the BPA degradation rate during PMS oxidation with a Sr_2_CoFeO_6_ catalyst was much higher than that for the reactors without a catalyst additive (Hammouda et al., 2018). The proportion of PS/PMS in the oxidation system was also essential to pollutant degradation because the excess quality of PS can induce a reverse reaction, whereas the insufficient amount induces a low removal rate. The work of Li et al. (2019b) found that the increasing dosage of PS from 1 to 10 mmol/L accelerated the decolorization of reactive brilliant blue (RBB) from 50.42 to 93.75%; however, the excess PS consumed the generated ${\text{SO}}_{4}^{-}\cdot$, via the following reactions (detailed in Equations 37–38), and led to a significant declining of the overall removal rate of RBB.

(37) $$\begin{array}{l} SO_{4}^{-}\cdot +SO_{4}^{-}\cdot \to S_{2}O_{8}^{2-} \end{array}$$

(38) $$\begin{array}{l} SO_{4}^{-}\cdot +S_{2}O_{8}^{2-}\to S_{2}O_{8}^{2-}+S_{2}O_{8}^{-}\cdot \end{array}$$

Furthermore, the categories of substances also influenced the degradation rate. Under the same experimental conditions (temperature and solution pH), the four typical antibiotics exhibited a decreasing trend—sulfisoxazole (100%) > sulfamethoxazole (98%) ≈ sulfathiazole (97%) > sulfamethizole (81%)—during the oxidation of PS/PMS (Zhou et al., 2019) (see Table 2).

### Degradation Intermediates of Different Pollutants After PS/PMS Oxidation

Substitution, decarboxylation, destruction, coupling reaction, etc., accompanied by PS/PMS oxidation, have been shown to lead to distinct intermediate products during contaminant degradation. For instance, the oxidation of bisphenol F resulted in significant production of intermediates of bis(4-hydroxyphenyl)methanol, 4,4′-dihydroxybenzophenone, and 4-hydroxyphenyl 4-hydroxybenzoate as the PS/PMS oxidation progressed (Hammouda et al., 2018). The category of the intermediates is closely related to the degradation pathway and reaction conditions. Wu et al. (2020) found that the molecular weights of the oxidative intermediates of BPA within the Fe_2_Co_1_-LDH/PS system decreased significantly when the reaction time increased from 10 to 30 to 60 min. The addition of different catalytic components also significantly affects the intermediate products; for instance, the intermediates of BPA in the Fe_3_O_4_/coal fly ash/PS system were different from those obtained from the CoS@GN-60/PMS system (Zhu et al., 2019). In contrast, the intermediates of BPA in the BC-nZVI/PS system were phenol, p-hydroquinone, 4-isopropenylphenol, and 4-hydroxyacetophenone (Liu et al., 2018a); these are similar to those in the γ-Fe_2_O_3_@BC/PS system (Rong et al., 2019). Typical intermediates and their structural characteristics of the different containments during the PS/PMS oxidation are summarized in Table 3.

**Table 3:** Chemical and structural characteristics of the intermediates of different pollutants during the PS/PMS oxidation.

**Target pollutant**	**Catalytic system**	**Intermediates category and structure**			**References**
		**Open-chain compounds**	**Aromatic compounds**	**Heterocyclic compounds**	
Carbamazepine	LaCoO_3_/PMS		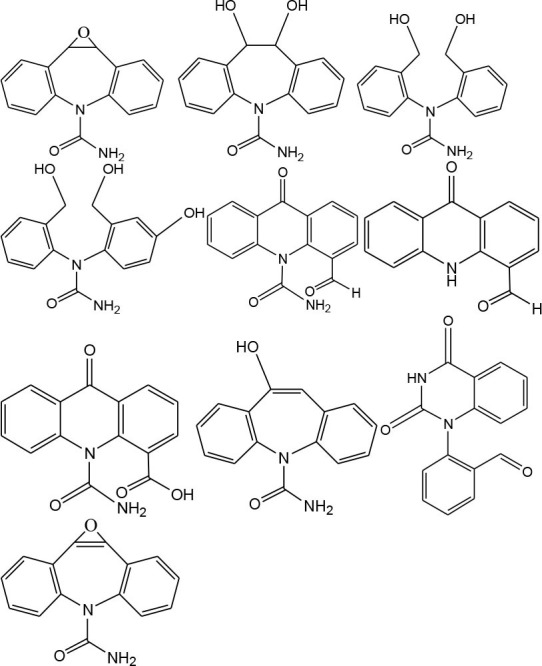		Guo et al., 2020
Levofloxacin hydrochloride (LVF)	CoFeO_2_@CN/PMS		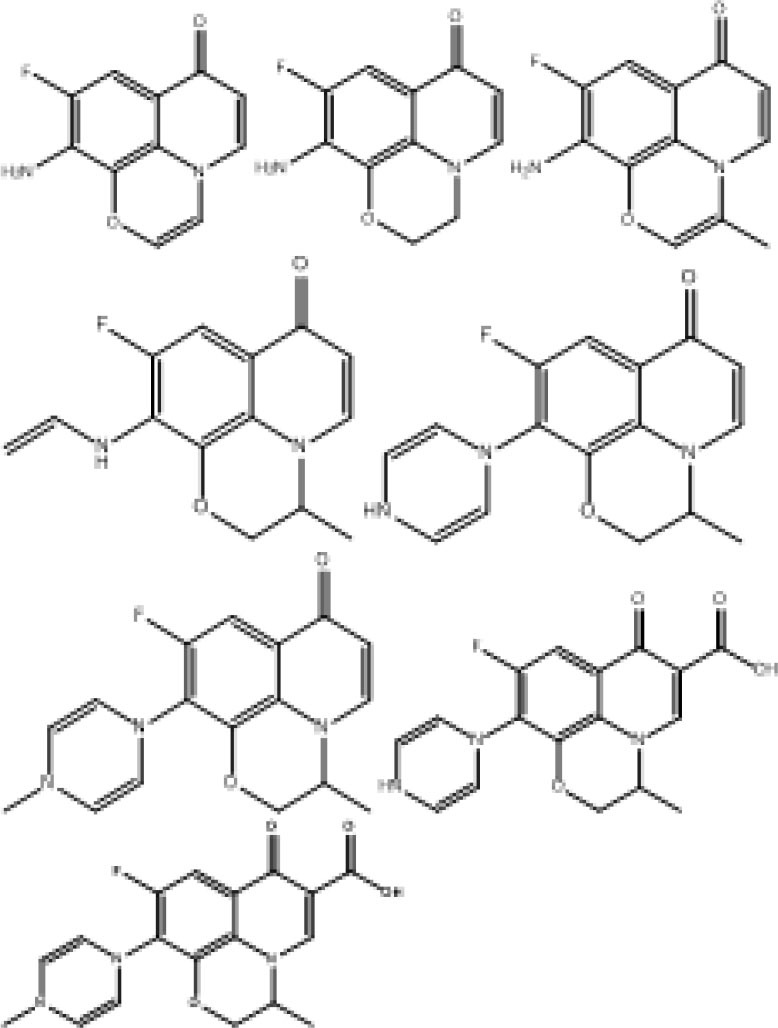		Pi et al., 2020
Ketoprofen (KET)	Heat/PS		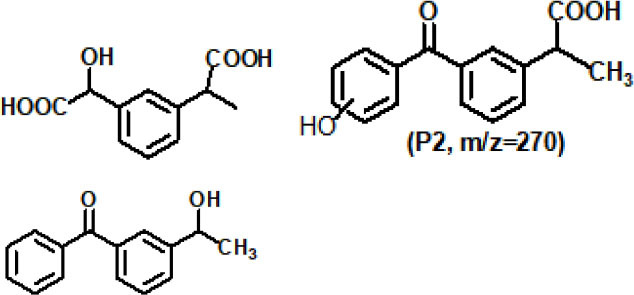		Feng et al., 2017b
Caffeine (CAF)	Co-CM41/PMS	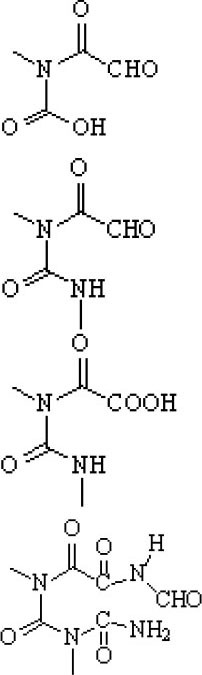		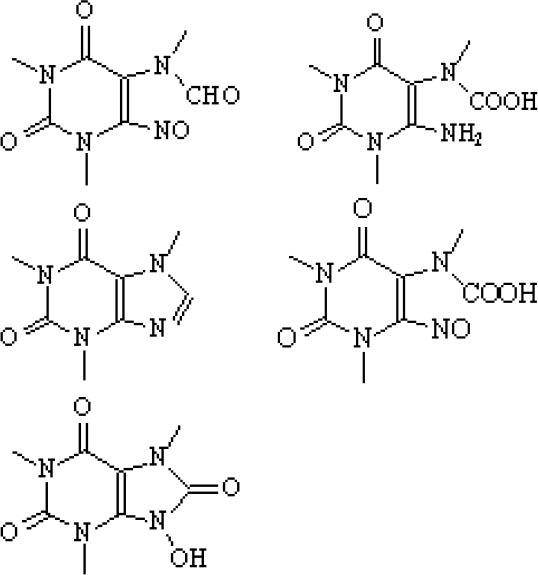	Qi et al., 2013
Diclofenac (DCF)	Bismuth ferrite (BFO)/PMS		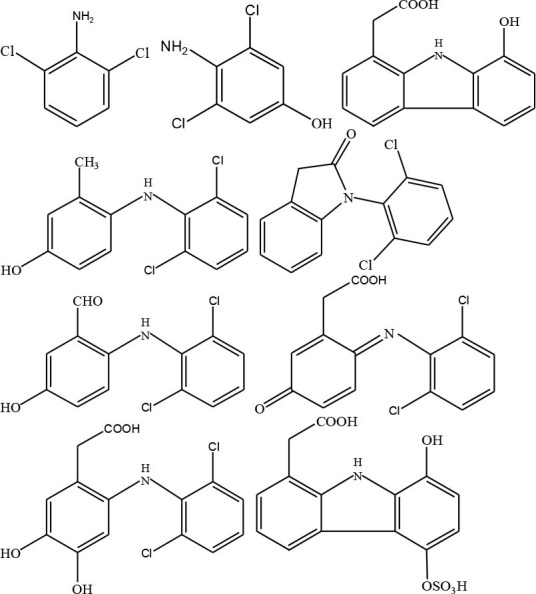		Han et al., 2020
Bisphenol A (BPA)	ZVI/PS	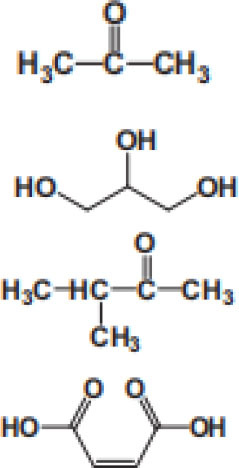	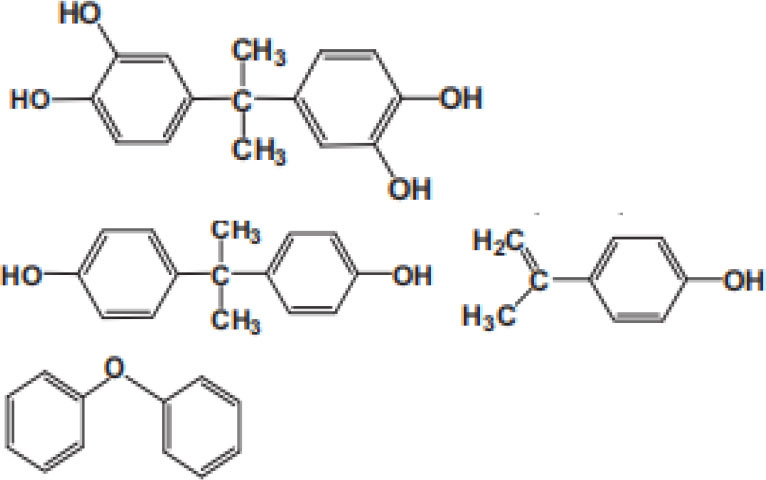		Zhao et al., 2016
Bisphenol F (BPF)		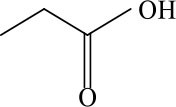	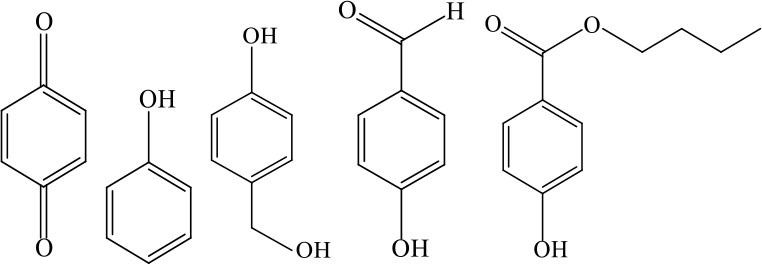		Hammouda et al., 2018

Experiments analyzing toxicity demonstrated that the majority of intermediates were less toxic than their parent pollutant. For example, the toxicity of the detectable intermediates of metolachlor (MET) (Liu C. et al., 2019), landfill leachate (Ghanbari et al., 2020), and carbamazepine (CBZ) (Guo et al., 2020) decreased significantly after PS/PMS oxidation. In contrast, the toxicity of intermediates produced from the degradation of highly concentrated organic wastewater increased significantly because of the formation of some halogenated organics (Wang Q. et al., 2017; Wang et al., 2020); the toxicity of the intermediates of alachlor was comparable to the formation of DBPs after chlorination within the ZVI/PS system (Wang et al., 2020). Similarly, Zhao et al. (2020) found that the formation of chloronitrophenols during thermally activated PS oxidation increased the toxicity of the intermediates during the oxidation of 2-chlorophenol in the presence of nitrite (${\text{NO}}_{2}^{-}$) (see Table 3).

## Potential Degradation Mechanisms of PS/PMS Oxidation

### Sulfate and Other Radical Oxidation Mechanisms

**Figure 2:**
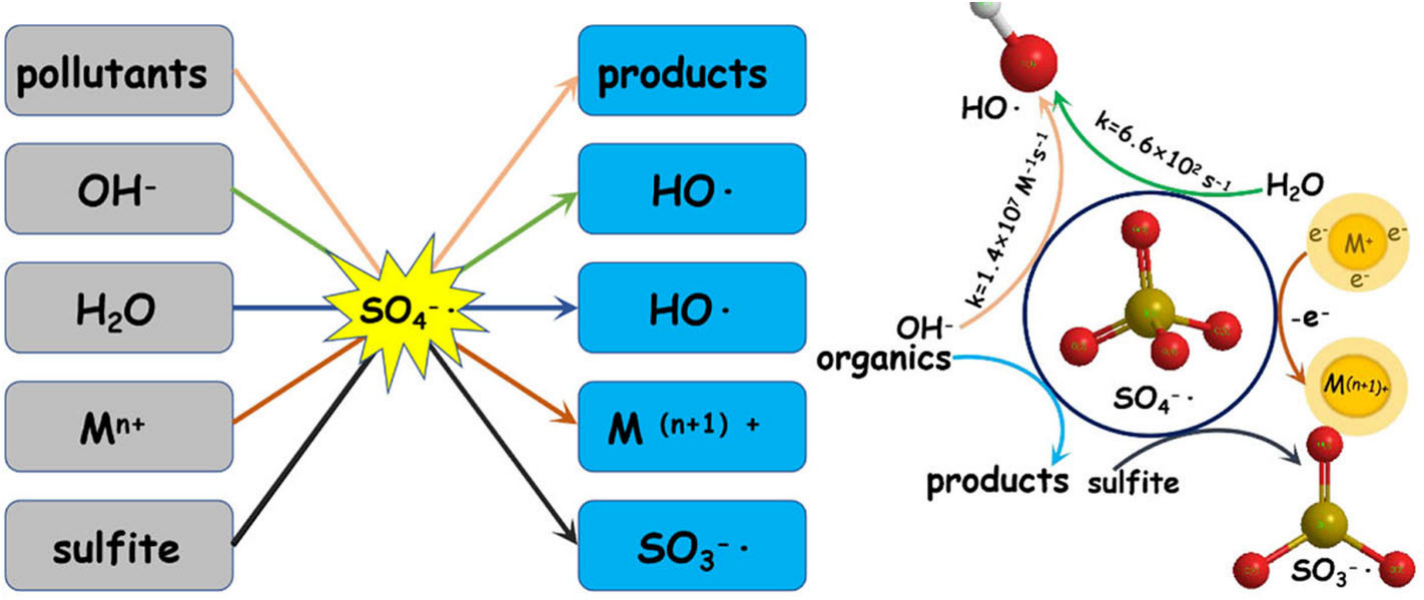
The role of sulfate radical in the sulfate-radical-based advanced oxidation process (SR-AOP) systems.

Sulfate radicals are critical to the PS/PMS oxidation mechanism; in particular, the kinetic characteristics of the reaction are essential for improving oxidation efficiency. The sulfate radical plays a significant role in pollutant oxidation via the SR-AOP system (see Figure 2 for a summary of the potential mechanisms).

In the PS/PMS system, the degradation efficiency of ${\text{SO}}_{4}^{-}\cdot$ is closely related to the chemical properties of the substrates. For most free radical reactions, ${\text{SO}}_{4}^{-}\cdot$ is the primary reactive oxygen species, and its oxidation ability is 10^4^ times that of ${\text{SO}}_{5}^{-}\cdot$ (Zhou et al., 2018). Specifically, ${\text{SO}}_{4}^{-}\cdot$ can provide electrons to attack the unsaturated bond (–N=C–) of the target pollutant of caffeine (CAF), to form intermediates (Qi et al., 2013). However, the destruction of the benzene ring by ${\text{SO}}_{4}^{-}\cdot$ via hydrogen abstraction is the main pathway for BPA degradation (Zhao et al., 2016).

In addition to the organic substrates, the SO_4_^**−**^**·** generated can also react within aqueous solutions with sulfite and transition metal ions, as well as itself. Specifically, the most noteworthy sacrificial path of the generated SO_3_^**−**^**·** pool is the reaction between SO_4_^**−**^**·** and sulfite; the latter thus has a complicated role in SR-AOPs. Theoretically, increasing the sulfite concentration would improve the substrate oxidation efficiency. However, excessive sulfite will scavenge ${\text{SO}}_{4}^{-}\cdot$ and inhibit substrate oxidation. The concentration and behavior of SO_4_^**−**^**·** can be analyzed via radical trapping experiments and electron paramagnetic resonance detection (Liu Y. et al., 2019; Wu et al., 2020). Further oxidation of H_2_O or OH^−^ by SO_4_^**−**^ leads to the generation of OH**·** (Equations 39–40), which would be another key reason for pollutant oxidation. The ${\text{SO}}_{4}^{-}\cdot$ and OH**·** produced can oxidize organic pollutants to different intermediates or inorganics through various degradation pathways (Hammouda et al., 2018). For example, Qi et al. (2013) found that the generated ${\text{SO}}_{4}^{-}\cdot$ and OH**·** radicals, with the Co^2+^/PMS system, can not only efficiently degrade caffeine but also mineralize the corresponding intermediates (see Figure 2).

(39) $$\begin{array}{l} SO_{4}^{-}\cdot +H_{2}O\to SO_{4}^{2-}+OH\cdot +H^{+} \end{array}$$

(40) $$\begin{array}{l} SO_{4}^{-}\cdot +OH^{-}\to SO_{4}^{2-}+OH\cdot \end{array}$$

The high concentration of sulfates, generated from the PS/PMS activation processes and side reactions, posed a potential threat to aquatic life (limited concentration of sulfate is 500 mg/L). However, the production and discharging of the sulfates during the PS/PMS activation are seldom focused. The recent studies revealed that one of the efficient approaches to overcome this limitation is the development of integrating the SR-AOP technique with cheaper oxidants, e.g., hydrogen peroxide or ozone (Duan et al., 2020). For example, Yang et al. (2015) proposed that the generated sulfates could be efficiently oxidated by the reaction of ozone with PMS.

### Kinetic Characteristics of SR-AOPs Used in Organic Degradation

SR-AOPs are complicated physicochemical processes; studying the thermodynamic and kinetic characteristics of the reaction of different pollutants is essential for clarifying the mechanisms. Generally, the pseudo-first-order (Equation 39) and pseudo-second-order kinetics (Equation 40) have been widely used for the simulation of the degradation reaction of organic compounds; these can be described by linear forms in Equations (41) and (42), respectively.

(41) $$\begin{array}{l} \text{ln}\frac{C_{t}}{C_{0}}=-k_{1}t \end{array}$$

(42) $$\begin{array}{l} \frac{1}{C_{0}}-\frac{1}{C_{t}}=-k_{2}t \end{array}$$

Where *C*_0_ is the initial concentration of the pollutant, *C*_*t*_ represents the pollutant concentration at a certain reaction time (*t*), and *k*_1_ and *k*_2_ are the pseudo-first-order and pseudo-second-order rate constants, respectively.

The respective species and chemical structures of organic pollutants significantly affect the kinetic characteristics of degradation. Some of the latest literature published revealing the kinetic mechanisms of typical organic pollutants during SR-AOPs is presented in Table 4. Overall, the degradation processes of (1) typical EDCs (such as bisphenol A, bisphenol F, and tetrabromobisphenol A) (Ji et al., 2016b; Hammouda et al., 2018; Xu et al., 2019) and (2) PPCPs (such as ketoprofen, ibuprofen, carbamazepine, etc.) (Feng et al., 2017; Wang et al., 2019; Guo et al., 2020) fit well with the pseudo-first-order kinetics. In contrast, the recent work of Lutze et al. (2015) found that the pseudo-second-order model yielded a much better fit for simulating the oxidation process of the chlorotriazine pesticides (such as atrazine, desethylterbuthylazine, and terbuthylazine).

**Table 4:** Kinetics model and rate constants of organic pollutants via SR-AOPs.

**Pollutants**	**Kinetics**	**Rate constants**	**References**
Ketoprofen	Pseudo-first-order	0.38 min^−1^	Feng et al., 2017
SCP	Pseudo-first-order	0.46 ± 2.3 × 10^−3^ min^−1^	Kang et al., 2019
Ibuprofen	Pseudo-first-order	0.0175 min^−1^	Wang et al., 2019
CBZ	Pseudo-first-order	0.26 min^−1^	Guo et al., 2020
Sulfonamides	Pseudo-first-order		Zhou et al., 2019
DEA	Pseudo-second-order	2.11 × 10^−3^ cm^2^mJ^−1^	Khan et al., 2017
DIA	Pseudo-second-order	4.6 × 10^−3^ cm^2^mJ^−1^	Khan et al., 2017
Clopyralid	Pseudo-first-order	3.29 × 10^−2^ min^−1^	Yang et al., 2020
BPA	Pseudo-first-order	0.0556 min^−1^	Xu et al., 2020
BPS	Pseudo-first-order	0.0445 min^−1^	Liu Y. et al., 2019
BPF	Pseudo-first-order	0.026 min^−1^	Hammouda et al., 2018

It is interesting to note that the degradation of bisphenol S in a heat/PS system followed a pseudo-zero-order model (Wang Q. et al., 2017), whereas neither of the abovementioned zero-order, first-order, and pseudo-second-order models could be applied for the simulation of the degradation of diclofenac within the bismuth ferrite (BFO)/PMS system. Han et al. (2020) stated that the diclofenac degradation curve could be well-fitted to a double exponential expression, as shown in Equation (43).

(43) $$\begin{array}{l} \frac{C_{t}}{C_{0}}=a_{1}e^{-k_{1}t}+a_{2}e^{-k_{2}t} \end{array}$$

Where *C*_*t*_ and *C*_0_ are the diclofenac concentration (mM) at times *t* and 0, respectively; *a*_1_ and *a*_2_ represent fraction coefficients and *k*_1_ and *k*_2_ are the apparent rate constants (min^−1^) for the first and second types of diclofenac degradation, respectively.

The reaction rates of different organic pollutants differ widely. Taking the reaction rate constant obtained from pseudo-first-order kinetics, for example, the abovementioned PPCPs, EDCs, and HCOM decrease in a trend of PPCPs (10^−2^-10^−1^) ≈ EDCs (10^−2^-10^−1^) > HCOM (10^−4^-10^−3^). Specifically, the obtained *k*_1_ value of ketoprofen was 0.26 min^−1^ in the heat/PS system at 70°C, whereas it declined to 3.29 × 10^−2^ min^−1^ when the temperature decreased to 60°C. The order of magnitude of the reaction rate constants was about 10^−3^-10^−4^ for pseudo-second-order kinetics (Khan et al., 2017). In addition, the changes in the experimental conditions could also affect the reaction rate constant; for example, the *k*_1_ value of bisphenol A during the reaction significantly increased from 0.07 to 0.26 min^−1^, when the catalyst loading increased from 0.2 to 0.4 g/L (Rong et al., 2019).

The species of radicals generated during SR-AOPs would also significantly affect the decomposition rate constant of the target pollutants. Zhao et al. (2016) reported that the degradation rate constant of BPA could be attributed to both the reactions with ${\text{SO}}_{4}^{-}\cdot$ and OH**·**, respectively, which can be described by Equations (44).

(44) $$\begin{array}{l} k_{obs}=k_{SO_{4}^{-}\cdot }^{\prime }C_{SO_{4}^{-}\cdot }+k_{OH\cdot }^{\prime }C_{OH\cdot } \end{array}$$

Where *k*′ represents the second-order rate constant for the reaction of BPA with each individual oxidizing species. Similarly, Wang et al. (2016) also found that the rate constants for the reaction of OH**·** were higher than those for ${\text{SO}}_{4}^{-}\cdot$ during the degradation process of 2,4-di-*tert*-butylphenol. Moreover, Nasseri et al. (2017) found that the second-order rate constants of DEA [(6.42 ± 0.12) × 10^8^ M^−1^ s^−1^] and DIA [(1.70 ± 0.30) × 10^9^ M^−1^ s^−1^] under ${\text{SO}}_{4}^{-}\cdot$ oxidation were lower than those of OH· [*k*_DEA_ = (1.14 ± 0.09) × 10^9^ M^−1^ s^−1^, *k*_DIA_ = (2.22 ± 0.44) × 10^9^ M^−1^ s^−1^] (see Table 4).

### Possible Mechanism of SR-AOPs for Degradation of Different Organics

As the dominant radical, ${\text{SO}}_{4}^{-}\cdot$ oxidizes the pollutants primarily by the following mechanisms: (1) hydrogen abstraction, (2) electron transfer, and (3) addition or substitution reaction. Electron transfer is the predominant mechanism of the reaction between ${\text{SO}}_{4}^{-}\cdot$ and aromatic organics. Taking the SR-AOP degradation of, for example, ketoprofen (Feng et al., 2017), bisphenol F (Hammouda et al., 2018), and bisphenol A (Xu et al., 2019), the attack or destruction of the benzene ring by ${\text{SO}}_{4}^{-}\cdot$ might be the main reason for their degradation. By comparison, hydrogen abstraction could easily occur during ${\text{SO}}_{4}^{-}\cdot$ reaction with saturated organic compounds (e.g., alkanes, alcohols, organic acids, ethers, and esters) (Zhao et al., 2016). For alkene and alkyne organics, the single electrons of the sulfate radical would attack the unsaturated bonds, and addition or substitution would enhance the degradation.

**Figure 3:**
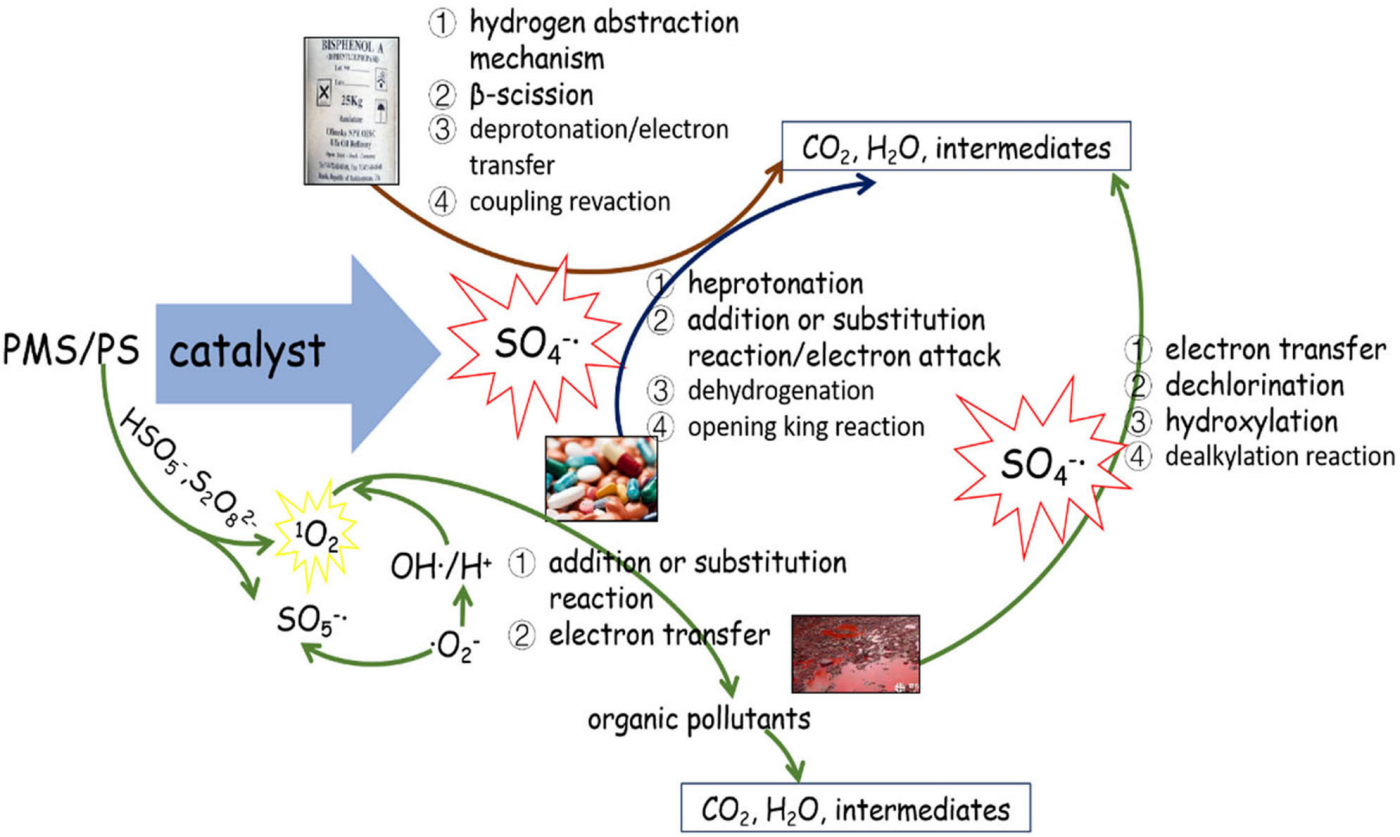
The possible mechanism of PS/PMS activation.

Generally, the concurrence of the abovementioned degradation pathways plays a significant role in the degradation of organic chemicals. For instance, the electrophilic attack of ${\text{SO}}_{4}^{-}\cdot$, hydroxylation, and coupling reactions were the main reasons for BPA oxidation within the ZVI/PS system (Zhao et al., 2016). In contrast, a deprotonate radical attacking an unsaturated bond in oxidizing and substitution reactions belonged to the CAF during the application of the Co-MCM41/PMS system (Qi et al., 2013). The non-free radical reaction was another reason for the partial pollutant degradation. For instance, the ^1^O_2_ generated from the self-decomposition of PMS (via Equation 45) would attack pollutants/intermediates via an electron transfer mechanism. According to the recent work of Liu Y. et al. (2019), ^1^O_2_ could easily be generated either from the reactions between **·**${\text{O}}_{2}^{-}$ and OH**·**/H^+^ or from direct formation from ${\text{SO}}_{5}^{-}\cdot$ (see Figure 3).

(45) $$\begin{array}{l} HSO_{5}^{-}+SO_{5}^{2-}\to HSO_{4}^{-}+SO_{4}^{2-}{+}^{1}O_{2} \end{array}$$

## Summary and Outlook

Sulfate radicals can be effectively generated through PS or PMS activation by physical methods (such as heating, UV, and ultrasound) and chemical methods (including transition metal ions, alkaline conditions), as well as coupling activation methods. Overall, SR-AOPs can not only be applied to the treatment of refractory organics in wastewater but also are efficient in dealing with novel/emerging pollutants such as PPCPs, antibiotics, and DBP precursors. Not only can toxic pollutants be destroyed during SR-AOPs, but also the toxicity of most of the intermediates can also be reduced. Given the multiple sources of pollutants within wastewater and the complex reaction conditions, difficulty in implementing SR-AOPs for wastewater treatment is a barrier to PS/PMS oxidation. Therefore, we recommend the following research directions:

1. Prioritize research on the treatment/degradation of multiple pollutants using SR-AOP oxidation; in the municipal wastewater treatment process, sewage is unlikely to contain only one target pollutant.
2. Develop novel and effective catalysts that can be used to upgrade the SR-AOP system to not only target pollutant directly but also efficiently improve the degradation efficiency of the pollutants.
3. Despite strong research proving that SR-AOPs are efficient at removing organic pollutants, dealing with wastewater at a large scale is still the main challenge. Therefore, a further direction is the search for alternative activation approaches that are economical and environment-friendly and can be applied in large-scale WWTPs.
4. Most researchers have focused on how to efficiently generate free radicals, but seldom on free radical or non-free radical degradation pathways for organics.
5. Research on the properties of intermediates, especially their toxicity, needs greater clarity. Thus, determining the fate of pollutants and their intermediates across their complete life cycle during SR-AOP oxidation should be prioritized.

## Author Contributions

All authors listed have made a substantial, direct and intellectual contribution to the work, and approved it for publication.

## Conflict of Interest

The authors declare that the research was conducted in the absence of any commercial or financial relationships that could be construed as a potential conflict of interest.
